# WeatherMamba: Reliable Geometry-Aware Generalization for Adverse-Weather LiDAR Sensor Point-Cloud Semantic Segmentation

**DOI:** 10.3390/s26185691

**Published:** 2026-09-08

**Authors:** He Huang, Xintai Zhang, Yidan Zhang, Junxing Yang

**Affiliations:** School of Geomatics and Urban Spatial Informatics, Beijing University of Civil Engineering and Architecture, Beijing 102616, China; huanghe@bucea.edu.cn (H.H.); 2108570425087@stu.bucea.edu.cn (X.Z.); 2108570424023@stu.bucea.edu.cn (Y.Z.)

**Keywords:** LiDAR sensors, point-cloud semantic segmentation, adverse-weather perception, domain generalization, state-space model, autonomous driving

## Abstract

Adverse weather degrades LiDAR semantic segmentation by altering return density, measured intensity, local geometry, and feature reliability. We study source-only domain generalization from clear-weather or synthetic training data to unseen real adverse-weather scenes. WeatherMamba is a geometry- and reliability-aware state-space framework that integrates multiscale neighborhood aggregation, reliability-conditioned feature refinement, and efficient long-range sequence modeling. Its central component, Weather-Guided Reliability Gating (WGRG), derives a continuous point-wise gate from conditionally normalized measured intensity, multiscale local density, and latent features. This gate bounds residual corrections without an externally supplied weather type, prior target-reflectance values, or deployment-time meteorological measurements. A controlled pseudo-weather strategy models signal perturbation and point-support loss separately during source-domain training. WeatherMamba achieves 37.4% mIoU on SemanticKITTI→SemanticSTF and 22.8% on SynLiDAR→SemanticSTF, ranking third overall while attaining the highest class IoU in five classes under each protocol. In the single-seed module ablation, the complete configuration improves the module-free baseline from 29.4% to 37.4%. Relative to PTv3 with Modules, WeatherMamba reduces parameters, FLOPs, latency, and peak allocated memory by 19.1%, 34.8%, 16.5%, and 25.2%, respectively, with a 0.4-point reduction in mIoU. These results indicate a competitive accuracy–efficiency trade-off under the evaluated protocols.

## 1. Introduction

LiDAR point-cloud semantic segmentation assigns a semantic label to each 3D point and supports localization, mapping, obstacle reasoning, and motion planning in autonomous-driving systems. Although current networks perform well on clear-weather benchmarks, their reliability can deteriorate in rain, fog, and snow. Controlled sensor studies show that adverse weather changes the number of valid returns, measured intensity, target visibility, and detection range. Increasing rain can reduce both detected-point counts and measured return intensity [1,2]. Fog and rain also reduce automotive LiDAR target-detection performance [3,4], while controlled snow experiments report attenuation, backscattering, false detections, and sensor-surface contamination [5]. These effects alter the geometric and radiometric evidence available to a segmentation network, creating a distribution shift between source-domain training data and adverse-weather deployment data.

**Figure 1:**
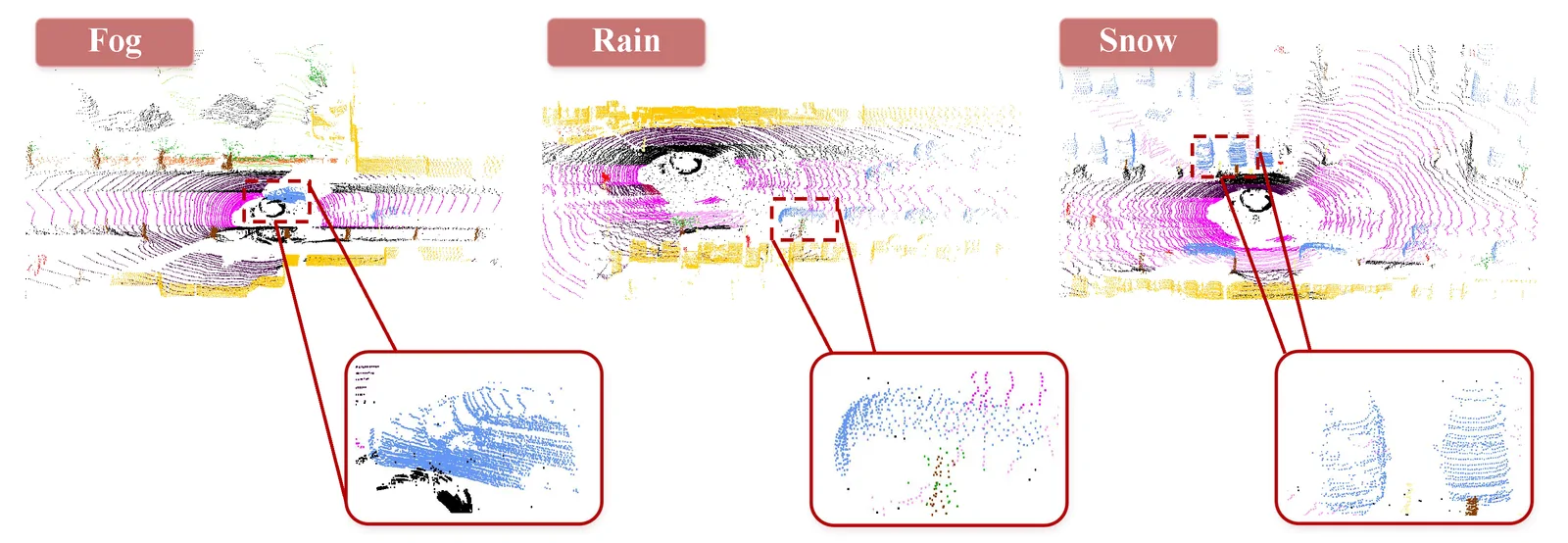
Effects of fog, rain, and snow on SemanticSTF point clouds. Point colors denote semantic classes, and red boxes indicate enlarged local regions.

Figure 1 illustrates representative SemanticSTF [6] point clouds under fog, rain, and snow conditions.

We consider domain generalization rather than target-domain adaptation. The model is trained on clear-weather or synthetic source-domain data and evaluated directly on unseen real adverse-weather scenes. No SemanticSTF target-domain frame is used for model optimization. This setting reflects the practical difficulty of collecting and labeling every combination of sensor, road scene, precipitation level, visibility, and target material. A deployed autonomous system must, nevertheless, remain reliable when weather conditions differ from those represented during training.

Adverse-weather generalization presents several coupled difficulties. Attenuation and point loss weaken local geometric continuity, especially around thin structures, object boundaries, and distant targets. Sparse artifacts and unstable returns can produce unreliable local features that are then amplified by deeper layers. When local evidence is incomplete, recognition also depends more strongly on long-range context. Degradation also varies continuously and cannot be represented adequately by a simple light/heavy label. A practical model should, therefore, infer degradation cues from the observed point cloud while preserving local geometry and long-range context.

Recent methods address different aspects of adverse-weather LiDAR domain generalization. Weather-Aware Autopilot introduces adaptive normalization, dual-attention fusion, and proxy-label learning for SemanticKITTI/SynLiDAR→SemanticSTF transfer [7]. Park et al. analyze geometric perturbation and point drop and design targeted augmentation for adverse-weather segmentation [8]. No Thing, Nothing addresses the disproportionate degradation of safety-critical “things” classes through superclass binding and beam-wise feature distillation [9]. PhyDAWS uses physically inspired weather simulation for domain-generalized point-cloud segmentation [10]. Together, these studies support feature adaptation, targeted augmentation, safety-critical class modeling, and physically inspired simulation. Observation-derived continuous point-wise degradation, explicit local geometric support, and efficient long-range context, however, have not been studied jointly.

We address this gap with WeatherMamba, a geometry- and reliability-aware state-space framework for adverse-weather LiDAR segmentation. Its central component, WGRG, represents degradation as an observation-derived continuous point-wise gate, not as a discrete weather-class decision or a physically calibrated severity estimate. WGRG combines measured intensity, multiscale local density, and latent-feature evidence to bound residual corrections using only the observed scan and source-trained quantities at inference. MANF aggregates multiscale neighborhoods to preserve local geometric support, RADM refines features according to point-wise reliability without removing points, and a hierarchical selective state-space backbone propagates long-range context efficiently. During training, weather-motivated augmentation exposes the source model to controlled signal perturbation and support-loss patterns. Each component, therefore, addresses a distinct failure mode within the same source-only framework.

We evaluate two source-only transfer protocols using SemanticKITTI or SynLiDAR as the source and SemanticSTF as the common unseen target. The experiments report aggregate, class-wise, and weather-wise accuracy; complete module combinations; WGRG evidence sensitivity; pseudo-weather strength sensitivity; and a matched PTv3 [11] efficiency comparison. This evaluation examines both segmentation accuracy and the computational cost of observation-only deployment.

Our main contributions are summarized as follows:

1. WeatherMamba integrates MANF, RADM, and a hierarchical Mamba backbone to address local geometric support, feature reliability, and long-range context within a unified source-only architecture.
2. WGRG derives a continuous point-wise gate from measured LiDAR intensity, multiscale density, and latent-feature evidence. It bounds residual corrections without an external weather label, prior target-reflectance values, or deployment-time meteorological input.
3. A weather-motivated augmentation strategy controls signal perturbation and point-support loss separately. Pre-specified default strengths are evaluated through a post hoc sensitivity analysis over neighboring settings.
4. Under two SemanticSTF transfer protocols, the complete configuration improves the module-free baseline by 8.0 points. Relative to PTv3 with Modules, it reduces parameters, FLOPs, latency, and peak memory with a 0.4-point mIoU trade-off.

## 2. Related Work

### 2.1. LiDAR Semantic Segmentation and Long-Range Context Modeling

LiDAR semantic segmentation methods are commonly organized into point-based, projection-based, voxel-based, and global-context architectures. PointNet++ [12] and RandLA-Net [13] learn hierarchical local features directly from point sets. Projection-based methods, including SqueezeSegV3 [14] and SalsaNext [15], convert point clouds into range-view representations for efficient 2D processing. Voxel-based methods such as Cylinder3D [16], PolarNet [17], and sparse-convolution networks regularize 3D space and achieve strong outdoor segmentation accuracy. Projection and voxelization, however, can compress or quantize fine geometry, while repeated neighborhood aggregation can be costly for long outdoor scans.

Global context becomes especially important when adverse weather removes or corrupts local evidence. Transformer-style self-attention captures long-range dependencies but scales quadratically with sequence length. Structured state-space models offer a more efficient alternative. S4 [18] and Mamba [19] support efficient long-sequence modeling, while Vision Mamba [20] and VMamba [21] adapt selective state-space propagation to visual data. In point clouds, however, serialization can separate physically adjacent points. WeatherMamba, therefore, combines state-space propagation with explicit multiscale neighborhood recovery.

### 2.2. Physical Effects of Adverse Weather on LiDAR Measurements

Sensor-level studies document the local geometry degradation, unstable returns, and measured-intensity shifts introduced by adverse weather. Filgueira et al. [1] reported that increasing rain can reduce LiDAR intensity and the number of detected points. Goodin et al. [2] modeled rain-rate-dependent degradation for automotive LiDAR, and Kutila et al. [3] measured substantial performance loss in controlled fog and rain. Montalban et al. [4] compared automotive LiDAR point clouds in artificial rain and fog, showing that degradation depends on both sensing technology and environmental conditions. Controlled snowfall experiments have also reported attenuation, backscattering, false detections, and sensor contamination [5]. These findings indicate that degradation varies continuously with atmospheric severity, range, sensor characteristics, and scene properties.

Throughout this paper, the per-point radiometric value provided by SemanticKITTI and SynLiDAR is termed measured LiDAR intensity (or remission), rather than intrinsic material reflectance. It depends on target properties, range, incidence angle, sensor calibration, and atmospheric propagation. WeatherMamba, therefore, requires no database of target-reflectance values. Measured intensity is used only as one observation-derived cue, together with point density and latent-feature statistics.

### 2.3. Adverse-Weather Domain Generalization and Data Augmentation

Early robustness strategies include geometric filtering and generic data augmentation. Radius/statistical outlier filtering and DROR-style approaches can suppress isolated weather returns but depend on hand-tuned thresholds and may also remove valid sparse structures [22,23]. PolarMix [24] improves LiDAR training diversity by mixing point-cloud sectors, while PointDR [6] regularizes source-domain representations for adverse-condition generalization. UniMix [25] and recent domain-generalization approaches further improve transferability through mixing, uncertainty, or representation learning.

More recent work explicitly targets adverse-weather domain generalization. Weather-Aware Autopilot [7] combines adaptive feature normalization, dual-attention fusion, and proxy-label generation, using SemanticKITTI and SynLiDAR as source domains and SemanticSTF as the unseen target. Rethinking Data Augmentation [8] identifies geometric perturbation and point drop as major data-level factors and proposes selective jittering and learnable point drop. No Thing, Nothing [9] shows that safety-critical “things” classes degrade disproportionately and introduces feature binding and beam-wise distillation. PhyDAWS [10] uses physically inspired weather simulation, including weather-specific attenuation and density/occlusion mechanisms, to improve domain-generalized point-cloud segmentation. These methods form the recent comparison context for WeatherMamba.

WeatherMamba complements these approaches by placing observation-derived point-wise gating within a geometry- and reliability-aware state-space architecture. Its pseudo-weather branch models sensor-relevant degradation directions but is not intended as an exact atmospheric simulator. The sensitivity analysis consequently reports the strongest settings only within the tested range.

### 2.4. Methodological Positioning

WeatherMamba coordinates long-range sequence modeling with dedicated local-geometry and reliability pathways and an observation-derived gate that requires no discrete weather metadata. The hierarchical Mamba backbone models long-range context, MANF aggregates multiscale local support, RADM performs reliability-conditioned refinement, and WGRG applies bounded point-wise recalibration using measured-intensity, density, and latent-feature evidence. The module and sensitivity studies evaluate these design choices under a fixed source-only protocol.

## 3. Materials and Methods

### 3.1. Overall Framework

**Figure 2:**
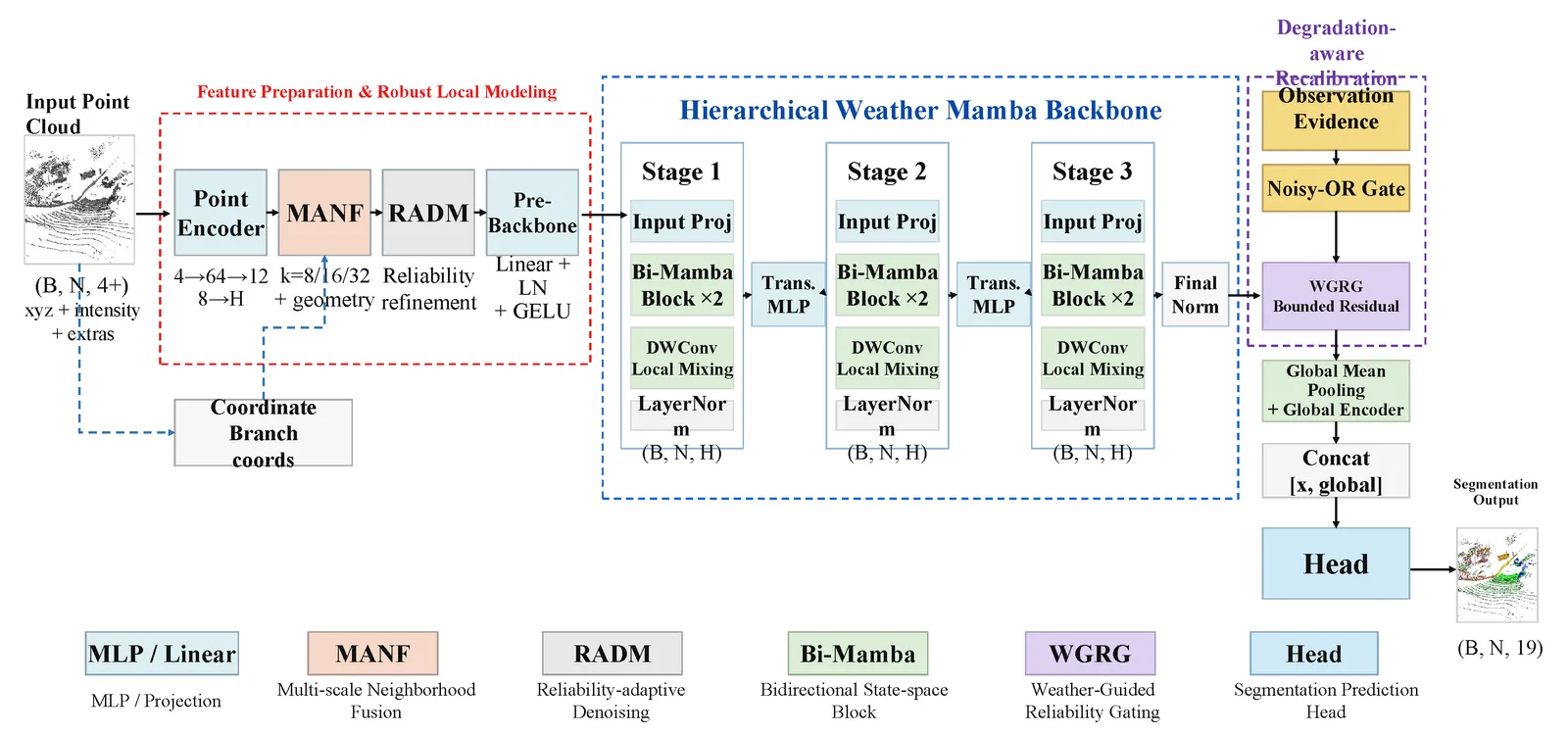
Overall architecture of WeatherMamba. Colors distinguish the functional components identified in the legend.

WeatherMamba performs semantic segmentation of LiDAR point clouds affected by weather-induced degradation. As shown in Figure 2, an input batch is represented as $P\in R^{B\times N\times D_{in}}$, where *B* is the batch size, *N* is the number of sampled points per scan, and $D_{in}$ is the per-point input dimension. Each point contains 3D coordinates (x,y,z), a measured LiDAR intensity/remission value, and optional auxiliary attributes. Measured intensity is treated as a sensor-return attribute, not as intrinsic, pre-known target reflectance.

The pipeline has four stages. Feature preparation first maps coordinates, measured intensity, and auxiliary attributes into a latent space. MANF aggregates multiscale neighborhoods to recover local geometric continuity, and RADM estimates point-wise reliability from local statistics to suppress unstable responses softly. The hierarchical WeatherMamba backbone then propagates the refined features through bidirectional selective state-space blocks to capture long-range context. WGRG derives a continuous degradation level for each point from measured-intensity, multiscale point-density, and latent-feature evidence. This level gates a bounded point-wise residual correction without a target-weather class label. Finally, the local and global representations are fused and mapped to point-wise semantic predictions.

### 3.2. Multiscale Adaptive Neighborhood Fusion

Point-cloud serialization enables efficient sequence modeling, but spatial neighbors may be separated in the resulting sequence. Under adverse weather, sparse returns, scattering noise, and partial occlusion further weaken local geometric continuity. The Multiscale Adaptive Neighborhood Fusion (MANF) module precedes the WeatherMamba backbone to recover local structure and provide geometric evidence.

**Figure 3:**
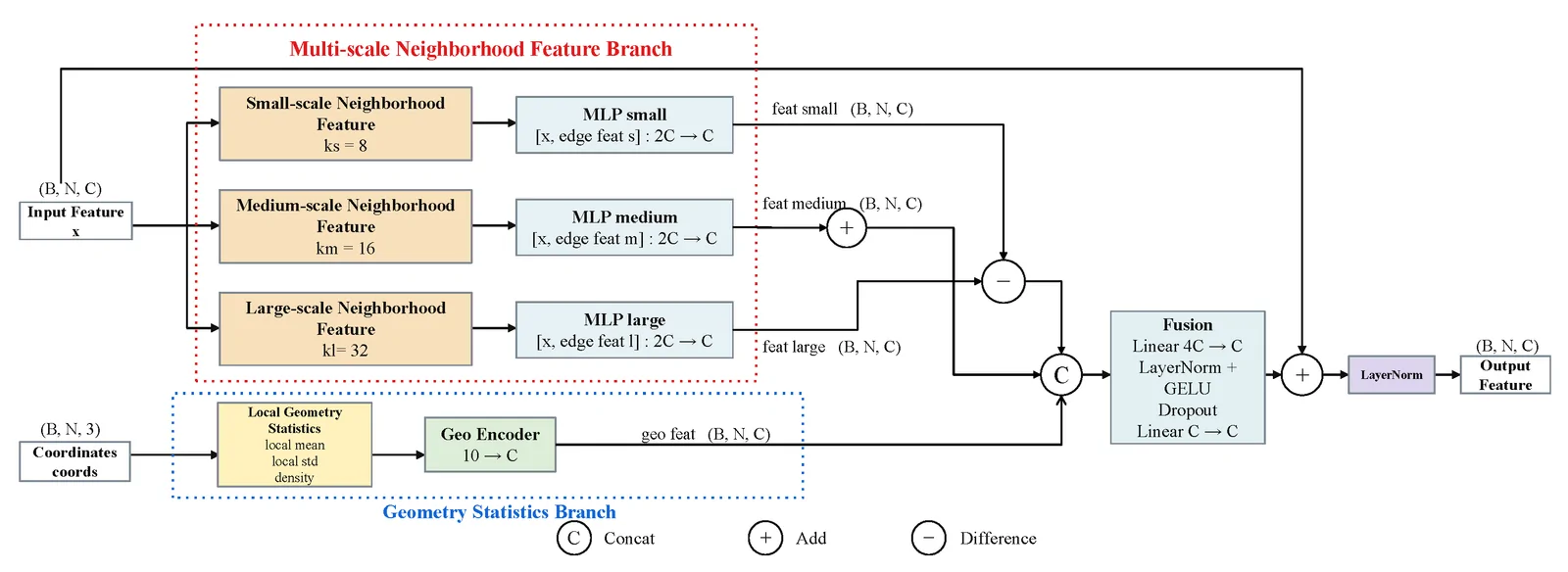
Architecture of the MANF module.

As illustrated in Figure 3, MANF receives point features $x\in R^{B\times N\times C}$ and coordinates $p\in R^{B\times N\times 3}$. For each point, the feature branch constructs three neighborhoods with $k_{s}=8$, $k_{m}=16$ and $k_{l}=32$. The edge feature at each scale is concatenated with the center feature and projected by a scale-specific MLP:

(1) $$F_{s}=\phi_{s}\left(\left[x,e_{s}\right]\right),\;F_{m}=\phi_{m}\left(\left[x,e_{m}\right]\right),\;F_{l}=\phi_{l}\left(\left[x,e_{l}\right]\right),$$

where $F_{s}$, $F_{m}$ and $F_{l}$ denote small-, medium- and large-scale neighborhood features. The three branches represent fine boundaries, intermediate structures, and the more stable geometry obtained from broader neighborhoods. The neighborhood sizes {8,16,32} form a compact geometric progression that covers these scales while keeping neighborhood construction tractable. They remain fixed across all reported experiments and SemanticSTF weather subsets. Here, *C* is the latent channel dimension of the current local block and is distinct from the number of semantic classes.

The multiscale branch explicitly models both aggregation and contrast across receptive fields. The additive multiscale context is defined as

(2) $$F_{sum}=F_{s}+F_{m}+F_{l},$$

while the cross-scale difference term is computed as

(3) $$F_{diff}=\left(F_{m}+F_{l}\right)-F_{s}.$$

The difference term emphasizes patterns that persist across larger neighborhoods while suppressing isolated small-neighborhood responses associated with weather-induced noise.

In parallel, the geometry statistics branch computes local mean, local standard deviation and density from the point coordinates. These statistics are projected by a geometry encoder to obtain $G_{geo}\in R^{B\times N\times C}$. The final fusion input is

(4) $$F_{cat}=x⊕F_{sum}⊕F_{diff}⊕G_{geo},$$

where ⊕ denotes channel concatenation. The output is generated by a fusion mapping followed by residual normalization:

(5) $$F_{out}=\mathrm{LayerNorm}\left(x+\psi (F_{cat})\right),$$

where ψ(·) consists of a linear projection, GELU activation, dropout, and a final linear layer. MANF, thus, restores local geometric context before global propagation by the sequence model.

### 3.3. Reliability-Aware Denoising Module

The Reliability-Aware Denoising Module (RADM) suppresses outlier responses in feature space. In contrast to hard-threshold preprocessing, RADM retains all points and estimates their reliability from local geometric statistics. It comprises local-statistics extraction, noise detection and feature refinement, and confidence-guided adaptive fusion.

**Local geometric statistics extraction:** For each point, RADM computes the local mean, standard deviation, point density, and feature-similarity variance within its *k*-nearest neighborhood. Object surfaces generally exhibit greater local continuity than sparse weather-induced returns. These statistics are concatenated with the original point features to provide structural cues for reliability estimation.

**Noise detection and feature refinement:** An MLP with sigmoid activation processes the original feature $F_{in}$ and the local statistics to estimate a point-wise noise probability $S\in {\left[0,1\right]}^{N\times 1}$. A larger $S_{i}$ indicates lower reliability. A parallel branch uses the local mean context to generate the candidate feature $F_{repair}=F_{in}+\mathrm{MLP}\left(F_{in},\mathrm{Mean}\right)$, where the residual connection preserves the original semantic information and improves training stability.

**Confidence-guided adaptive fusion:** The noise probability first interpolates between the original and refined features. ConfidenceNet then predicts a point-wise gate *G* $\in \;{\left[0,1\right]}^{N\times 1}$ from $F_{in}$ and *S*, controlling the contribution of the refined branch:

(6) $$\begin{array}{cc} F_{inter} & =\left(1-S\right)⊙F_{in}+S⊙F_{repair},\\ F_{out} & =G⊙F_{inter}+\left(1-G\right)⊙F_{in}. \end{array}$$

where ⊙ denotes element-wise multiplication. The two-stage weighting suppresses unreliable responses while retaining sparse but informative points, without requiring manual noise annotations.

### 3.4. Hierarchical WeatherMamba Backbone

The hierarchical WeatherMamba backbone models long-range dependencies with efficient computation. Selective state-space modeling has linear sequence complexity, in contrast to full self-attention, and is, therefore, suitable for large outdoor LiDAR scans. In the stage-wise design shown in Figure 2, each stage contains an input projection, two bidirectional Mamba blocks, depth-wise convolutional local mixing, and LayerNorm. Transition MLPs connect adjacent stages, and a final normalization layer produces the high-level representation processed by WGRG.

The unordered point cloud is first serialized according to its spatial coordinates. Each bidirectional Mamba block contains forward and backward scans. The forward branch processes the original sequence, while the backward branch processes its reversal to provide complementary context. Their output is

(7) $$X^{l}=\phi \left(\mathrm{Concat}\left({\mathrm{Mamba}}_{f}\left(X^{l-1}\right),\mathrm{Rev}\left({\mathrm{Mamba}}_{b}\left(\mathrm{Rev}\left(X^{l-1}\right)\right)\right)\right)\right),$$

where $X^{l}$ is the feature of the *l*-th stage, ${\mathrm{Mamba}}_{f}\left(\cdot \right)$ and ${\mathrm{Mamba}}_{b}\left(\cdot \right)$ denote the forward and backward state-space branches, Rev(·) denotes sequence reversal, and ϕ(·) denotes linear projection and nonlinear fusion.

State-space scanning propagates long-range information efficiently, but serialization can weaken local physical neighborhoods. Each stage, therefore, applies depth-wise convolutional mixing to restore short-range interactions in the latent sequence. The residual update is

(8) $${\tilde{X}}^{l}=\mathrm{LN}\left(X^{l-1}+\mathrm{DWConv}\left(X^{l}\right)\right),$$

where DWConv(·) denotes local depth-wise convolutional mixing and LN(·) denotes LayerNorm. Stacking three stages integrates local geometric detail with long-range semantic context while retaining efficient sequence modeling.

### 3.5. Weather-Guided Reliability Gating

**Figure 4:**
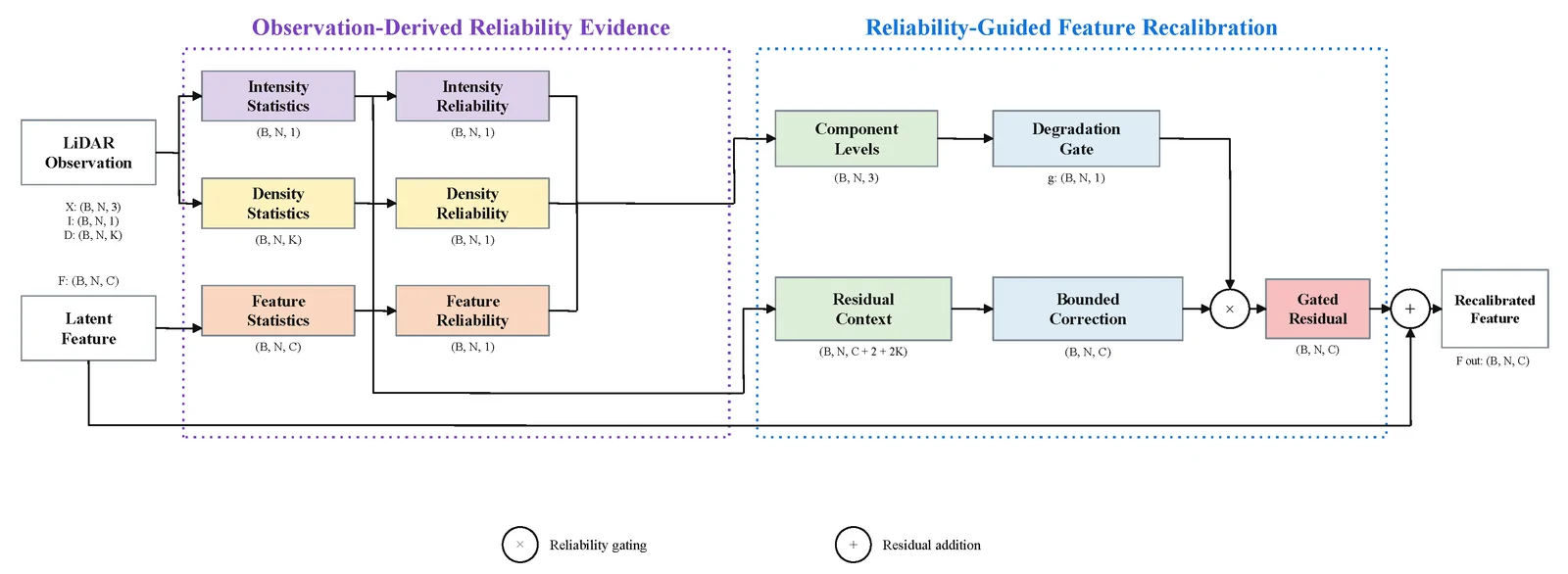
Architecture of the WGRG module.

Adverse-weather effects vary across points and are not represented reliably by a weather label assumed to be available at deployment. The Weather-Guided Reliability Gating (WGRG) module shown in Figure 4 instead models degradation as a continuous point-wise residual gate. Given point features $F={\left\{f_{i}\right\}}_{i=1}^{N}$ and $f_{i}\in R^{C}$, WGRG estimates $d_{i}\in \left[0,1\right]$ from measured LiDAR intensity, multiscale local density, and latent features. The resulting value bounds a residual correction. Intensity denotes sensor-provided intensity/remission rather than pre-known intrinsic material reflectance, and the scan-wise mean of $d_{i}$ is retained only for diagnostics.

A complete atmospheric model would require deployment-time measurements such as rain rate or visibility, as well as sensor-specific calibration and atmospheric parameters unavailable in the standard SemanticSTF inference setting. WGRG, therefore, estimates degradation from the observed point cloud while retaining physically motivated intensity and density cues. This design accommodates sensor-, range-, and scene-dependent effects without assuming meteorological inputs or a fixed mapping from weather intensity to point reliability.

Because intensity and density also vary with range, elevation, and sensor sampling, WGRG normalizes them using source-training medians *m* and median absolute deviations *a* conditioned on a 5×5 range/elevation grid. These statistics are frozen after fitting. Let b(i) denote the cell containing point *i*, and let $D_{i,k}$ denote its sensor-origin voxel occupancy at K=2 scales (0.2 and 0.4 m). The normalized evidence is

(9) $$z_{i}^{I}=\mathrm{clip}\;\left(\frac{\mathrm{asinh}\left(I_{i}\right)-m_{b(i)}^{I}}{\max(a_{b(i)}^{I},\varepsilon )},-8,8\right),\;z_{i,k}^{D}=\mathrm{clip}\;\left(\frac{\log\left(1+D_{i,k}\right)-m_{b(i),k}^{D}}{\max(a_{b(i),k}^{D},\varepsilon )},-8,8\right),$$

where $\varepsilon =10^{-5}$. Lightweight LayerNorm–GELU heads map the signed deviations and their magnitudes, together with the normalized latent feature, to component levels:

(10) $$\ell_{i}^{I}=\sigma (h_{I}([z_{i}^{I},|z_{i}^{I}|])),\;\ell_{i}^{D}=\sigma (h_{D}([z_{i}^{D},|z_{i}^{D}|])),\;\ell_{i}^{F}=\sigma \left(h_{F}\left(\text{LN}\left(f_{i}\right)\right)\right).$$

The feature component is learned without reference to a weather prototype. Noisy-OR combines the three components without a discrete weather decision:

(11) $$d_{i}=1-\left(1-\ell_{i}^{I}\right)\left(1-\ell_{i}^{D}\right)\left(1-\ell_{i}^{F}\right).$$

Inactive components are set to zero in the ablation, making $d_{i}$ equal to the component level when only one component is active.

The normalized feature and active physical evidence predict the correction direction, while $d_{i}$ controls its magnitude:

(12) $$u_{i}=\;[\text{LN}\left(f_{i}\right),z_{i}^{I},|z_{i}^{I}|,z_{i}^{D},|z_{i}^{D}\left|\right],\;c_{i}=\text{tanh}\left({\text{MLP}}_{res}\left(u_{i}\right)\right),\;f_{i}^{\prime }=f_{i}+\alpha d_{i}c_{i},\;\alpha =0.2.$$

Inactive intensity/density terms in $u_{i}$ are set to zero. Since $c_{i}\in {\left[-1,1\right]}^{C}$, each channel correction is bounded by $0.2d_{i}$. The final residual layer is initialized to zero, so WGRG begins as an identity mapping. WGRG, therefore, applies a point-wise scalar degradation gate to a point-wise channel correction, rather than using multiplicative channel attention.

The two occupancy scales represent fine and broader local support at modest computational cost. The 5×5 range/elevation partition balances condition specificity with the number of source samples available in each cell. The residual factor α=0.2 prevents WGRG from overwriting source-domain features, while the EMA momentum of 0.999 stabilizes the learned feature-evidence targets. Both settings remain fixed across the transfer protocols.

During training, corrupted and clean reference branches are generated from the same source scan and aligned by invariant point indices. For matched points, the targets are

(13) $$\begin{array}{cc} t_{i}^{I} & =\mathrm{clip}(|I_{i}^{c}-I_{i}^{r}|/128,0,1),\\ t_{i}^{D} & =K^{-1}\;\sum_{k}\mathrm{clip}\;\left(1-D_{i,k}^{c}/\left(D_{i,k}^{r}+10^{-6}\right),0,1\right),\\ t_{i}^{F} & =\mathrm{clip}\;\left((1-\cos\left(f_{T,i}^{c},f_{T,i}^{r}\right))/2,0,1\right), \end{array}$$

where $f_{T}$ is a stop-gradient exponential-moving-average feature teacher with momentum 0.999. For the active component set A, $t_{i}^{\left(A\right)}=1-\prod_{j\in A}\left(1-t_{i}^{j}\right)$. Let *V* contain the $N_{v}$ valid matches. The calibration, component, and clean-reference losses are

(14) $$\begin{array}{cc} L_{\mathrm{cal}} & =\frac{1}{N_{v}}\sum_{i\in V}SmoothL1\left(d_{i},t_{i}^{\left(A\right)}\right),\\ L_{\mathrm{component}} & =\frac{1}{3N_{v}}\sum_{i\in V}\sum_{j\in A}SmoothL1\left(\ell_{i}^{j},t_{i}^{j}\right),\\ L_{\mathrm{clean}} & =\frac{1}{N_{r}}\sum_{i}d_{i}^{r}. \end{array}$$

When WGRG is enabled, its supervision objective is

(15) $$L_{\mathrm{WGRG}}=0.5L_{\mathrm{cal}}+0.5L_{\mathrm{component}}+0.0L_{\mathrm{rank}}+0.05L_{\mathrm{clean}}.$$

Here, $L_{\mathrm{cal}}$ aligns the fused degradation score with the target formed from the active intensity, density, and feature evidence. $L_{\mathrm{component}}$ supervises the evidence-specific predictions, and $L_{\mathrm{clean}}$ suppresses degradation scores on clean reference points. $L_{\mathrm{rank}}$ is an optional ranking objective. Its weight is zero in the reported experiments, so it does not affect optimization. The equal 0.5 coefficients balance fused-degradation and component-level supervision, while the smaller 0.05 coefficient regularizes clean-reference scores without dominating the segmentation objective.

At inference, WGRG uses only the observed features, coordinates, measured intensity, multiscale density, frozen source-training statistics, and learned parameters. The paired reference, EMA teacher, supervision targets, and weather labels are used only during training. In Section 4.6.1, “intensity-only” and “density-only” refer to the evidence used to form $d_{i}$; the normalized latent feature remains common context for the correction direction. The combined intensity, density, and feature configuration gives the strongest result in the evidence study.

### 3.6. Weather-Motivated Pseudo-Weather Augmentation

Controlled pseudo-weather corruption is applied only to source-domain training scans to reduce the gap between clear/synthetic training data and unseen adverse-weather scenes. The augmentation follows sensor-relevant degradation directions supported by LiDAR studies and recent adverse-weather augmentation methods [1,3,4,8,10]: measured-intensity attenuation and perturbation, range-direction jitter, and loss of valid point support. It is not intended to reconstruct the complete atmospheric process. Separate sensitivity configurations use the field alpha with values in the range of {0,0.5,1.0,1.5}. We denote this discrete configuration value by *s* below; it is not supplied as a continuous run-time variable.

For a point with coordinate $x_{i}$ and measured intensity $I_{i}$, the support-preserving branch first applies linear intensity attenuation,

(16) $$I_{i}^{\mathrm{att}}=\left(1-a\left(s\right)\right)I_{i},\;a\left(s\right)=sa^{0},\;a^{0}\in \left\{0.15,0.30,0.45\right\}.$$

Impulse noise is then applied to a randomly selected subset of points. Let $M_{i}=⊮\left[U_{i}^{I}<q\left(s\right)\right]$, where $U_{i}^{I}∼U\left(0,1\right)$, $q\left(s\right)=sq^{0}$, and $q^{0}\in \left\{0,0.01,0.03\right\}$. The noise scale adapts to each scan:

(17) $$\sigma_{I,\mathrm{scan}}\left(s\right)=\max\;(\text{Std}\left[I^{\mathrm{att}}\right],10^{-3}),\;I_{i}^{\prime }=\max\;\left(0,I_{i}^{\mathrm{att}}+M_{i}z_{i}^{I}s\sigma_{I,\mathrm{scan}}\left(s\right)\right),$$

where $z_{i}^{I}∼N\left(0,1\right)$. The value *s* scales the attenuation rate, impulse probability, and conditional noise magnitude, while the absolute scale remains scan-adaptive.

Coordinate corruption follows the sensor-to-point ray rather than acting independently on the three coordinate axes. With $u_{i}=x_{i}/\max\left({∥x_{i}∥}_{2},10^{-6}\right)$, the perturbed coordinate is

(18) $$\delta_{i}\left(s\right)=\mathrm{clip}\;(z_{i}^{r}\sigma_{r}\left(s\right),-0.08s,0.08s),\;x_{i}^{\prime }=x_{i}+u_{i}\delta_{i}\left(s\right),$$

where $z_{i}^{r}∼N\left(0,1\right)$, $\sigma_{r}\left(s\right)=s\sigma_{r}^{0}$, and $\sigma_{r}^{0}\in \left\{0.005,0.015,0.03\right\}$ m. A shared per-axis preprocessing jitter with standard deviation 0.005 m, clipped to ±0.02 m, is applied before the clean/corrupted split. It is, therefore, not treated as weather degradation.

Point-support loss is controlled separately by a density/dropout strength d∈{0.5,1.0,1.5}. For $p_{\mathrm{drop}}^{0}\in \left\{0.05,0.15,0.30\right\}$, the configured probability is $p_{\mathrm{drop}}\left(d\right)=dp_{\mathrm{drop}}^{0}$. Point *i* is retained when $U_{i}^{D}\geq p_{\mathrm{drop}}\left(d\right)$, where $U_{i}^{D}∼U\left(0,1\right)$. A minimum-support safeguard retains at least 2048 points, so the realized drop ratio may be lower than the configured probability. The low, medium, and high candidates for attenuation, impulse probability, range jitter, and dropout are sampled independently with equal probability; they are not tied to the same level within a scan. In the support-preserving sensitivity panel, $p_{\mathrm{drop}}=0$ and s∈{0,0.5,1.0,1.5}. In the density/dropout panel, *s* is fixed at 0.5 and *d* is varied. SemanticSTF remains unseen during source-domain training, and both panels follow the evaluation protocol in Section 4.6.2.

### 3.7. Loss Function

The model is optimized on source-domain semantic labels using point-wise segmentation cross-entropy $L_{\mathrm{seg}}$. When WGRG is active, degradation-evidence supervision is added to this objective:

(19) $$L_{\mathrm{total}}=L_{\mathrm{seg}}+L_{\mathrm{WGRG}}.$$

The term $L_{\mathrm{seg}}$ uses only source-domain point labels, while $L_{\mathrm{WGRG}}$ is computed from paired corrupted/reference source scans, as defined above. Neither term uses SemanticSTF target-domain labels or class statistics.

## 4. Experiments and Results

WeatherMamba is evaluated under two adverse-weather domain-generalization protocols: SemanticKITTI→SemanticSTF and SynLiDAR→SemanticSTF. We report class-wise and weather-wise performance, module ablations, WGRG evidence sensitivity, pseudo-weather strength sensitivity, and computational efficiency.

### 4.1. Datasets and Metrics

SemanticKITTI [26] is the real clear-weather source domain. Following the standard split, sequences 00–07, 09, and 10 are used for training. SynLiDAR [27] is the synthetic source domain. SemanticSTF [6] is the unseen real-world target domain and includes dense fog, light fog, rain, and snow/sleet scenes. WeatherMamba is trained without any SemanticSTF frames.

Mean Intersection over Union (mIoU) over the 19 official semantic classes is the primary metric. Class-wise IoU and weather-wise mIoU are reported in percent (%). The sensitivity experiments additionally report mean class accuracy (mAcc) and overall point accuracy (allAcc). With K=19 classes, these metrics are defined as

(20) $$\begin{array}{cc} \mathrm{mAcc} & =\frac{1}{K}\sum_{k=1}^{K}\frac{{\mathrm{TP}}_{k}}{{\mathrm{TP}}_{k}+{\mathrm{FN}}_{k}},\\ \mathrm{allAcc} & =\frac{\sum_{k=1}^{K}{\mathrm{TP}}_{k}}{\sum_{k=1}^{K}\left({\mathrm{TP}}_{k}+{\mathrm{FN}}_{k}\right)}. \end{array}$$

The mAcc metric averages the accuracy of each class and, therefore, weights all classes equally. In contrast, allAcc is the proportion of correctly classified points over the full evaluation set and, therefore, reflects the class-frequency distribution.

### 4.2. Implementation Details and Evaluation Protocol

For SemanticKITTI→SemanticSTF, each method is trained on the SemanticKITTI source split and evaluated directly on SemanticSTF. For SynLiDAR→SemanticSTF, training uses SynLiDAR followed by evaluation under the same SemanticSTF target protocol. Results based on target-domain adaptation or different target splits are excluded because they are not directly comparable.

All experiments use a single NVIDIA GeForce RTX 4090 GPU, PyTorch 2.5.0, CUDA 12.4, and FP32 computation. Unless stated otherwise, WeatherMamba is trained for 80 epochs with AdamW, an initial learning rate of 0.001, weight decay of 0.01, and batch size of 4. Each scan is sampled or padded to 8192 points using the official 19-class mapping. Comparison methods are reproduced in-house from official implementations when available, or from the configurations described in their publications. Within each transfer direction, all methods use the same source and target splits, the official 19-class mapping, and a shared evaluation implementation while retaining their published optimization strategies and method-specific hyperparameters. No model is optimized with SemanticSTF labels.

Architectural and loss hyperparameters remain fixed across the two transfer protocols. MANF uses three neighborhood sizes, and the WGRG loss coefficients (0.5,0.5,0.0,0.05) are unchanged whenever the module is enabled. All numerical settings, including the default pseudo-weather strengths s=0.5 and d=1.0, are specified before target-domain evaluation and are not selected using SemanticSTF labels. The grid in Section 4.6.2 is a post hoc sensitivity analysis and does not determine the default configuration.

### 4.3. Comparison of Domain-Generalization Performance

Table 1 compares the class-wise IoU of WeatherMamba with representative domain-generalization methods. Table 2 reports the corresponding weather-wise mIoU.

Using SemanticKITTI as the source, WeatherMamba obtains 37.4% mIoU on SemanticSTF. This result is 0.8 percentage points below NTN [9] and 0.5 points below PhyDAWS [10]. Using SynLiDAR as the source, it obtains 22.8% mIoU and ranks third, behind UniMix [25] (23.4%) and PhyDAWS [10] (23.1%). It achieves the highest IoU for sidewalk, other-ground, fence, terrain, and pole under the first protocol, and for truck, bicyclist, parking, vegetation, and terrain under the second. These results cover both structural background classes and traffic participants. However, the aggregate mIoU remains below the strongest baselines in both protocols, indicating that the class-wise gains are not uniform.

**Figure 5:**
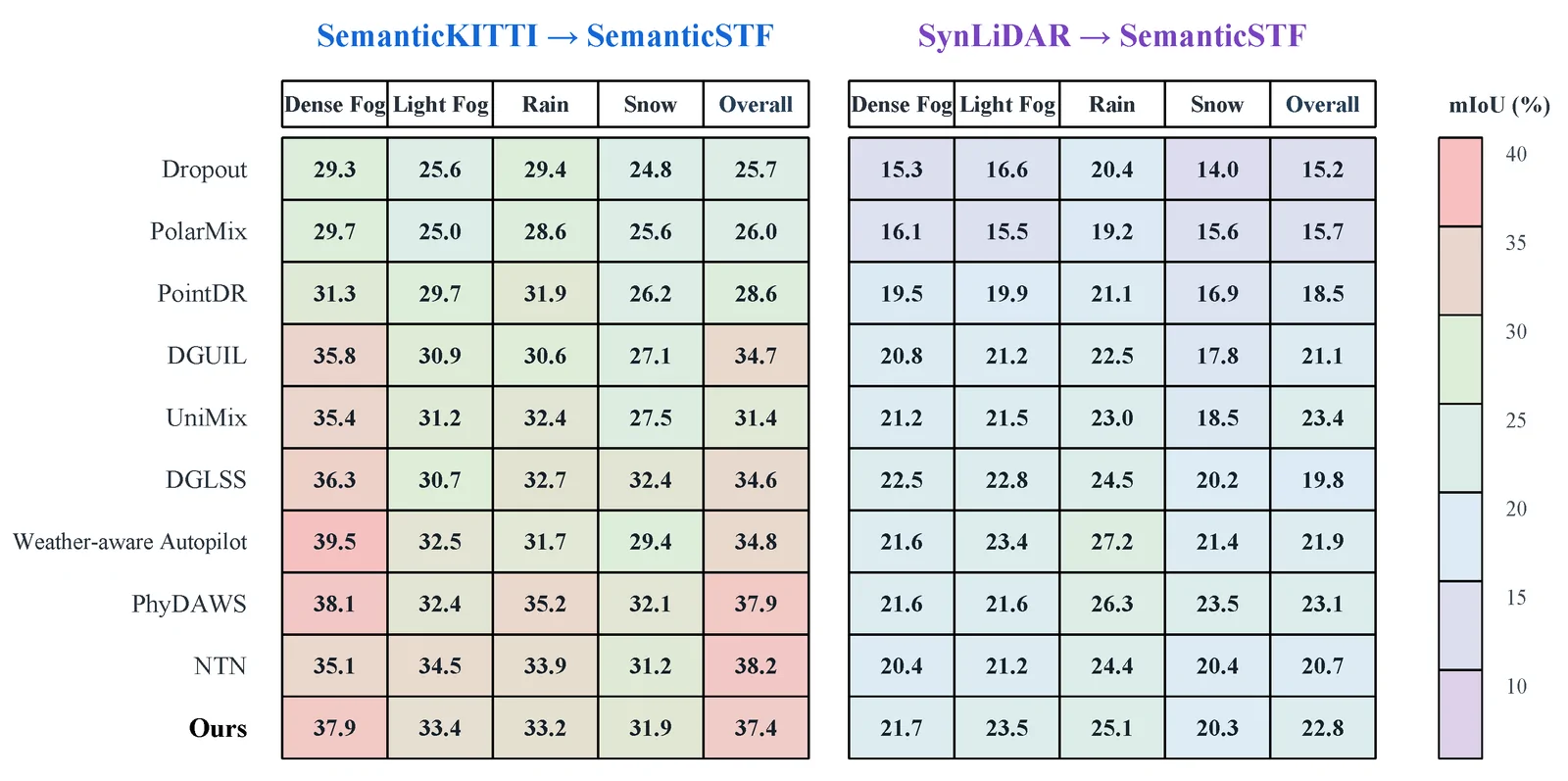
Weather-wise and overall mIoU comparison.

Figure 5 provides a compact weather-wise and overall comparison under both transfer protocols.

### 4.4. Weather-Wise Robustness

Table 2 reports the four weather-specific averages separately from the class-wise comparison. Panels (a) and (b) use SemanticKITTI and SynLiDAR as the source, respectively.

**Table 2:** Weather-wise mIoU (%) under the two domain-generalization protocols. The final mIoU column reports performance on the complete SemanticSTF validation set rather than the unweighted mean of the four weather subsets. The best and second-best results in each column are shown in bold and underlined, respectively.

(a) SemanticKITTI→SemanticSTF					
Method	D-fog	L-fog	Rain	Snow	mIoU (%)
Dropout [28]	29.3	25.6	29.4	24.8	25.7
PolarMix [24]	29.7	25.0	28.6	25.6	26.0
PointDR [6]	31.3	29.7	31.9	26.2	28.6
DGUIL [29]	35.8	30.9	30.6	27.1	34.7
UniMix [25]	35.4	31.2	32.4	27.5	31.4
DGLSS [30]	36.3	30.7	32.7	**32.4**	34.6
Weather-aware Autopilot [7]	**39.5**	32.5	31.7	29.4	34.8
PhyDAWS [10]	38.1	32.4	**35.2**	32.1	37.9
NTN [9]	35.1	**34.5**	33.9	31.2	**38.2**
**Ours**	37.9	33.4	33.2	31.9	37.4
**(b) SynLiDAR→SemanticSTF**					
Method	D-fog	L-fog	Rain	Snow	mIoU (%)
Dropout [28]	15.3	16.6	20.4	14.0	15.2
PolarMix [24]	16.1	15.5	19.2	15.6	15.7
PointDR [6]	19.5	19.9	21.1	16.9	18.5
DGUIL [29]	20.8	21.2	22.5	17.8	21.1
UniMix [25]	21.2	21.5	23.0	18.5	**23.4**
DGLSS [30]	**22.5**	22.8	24.5	20.2	19.8
Weather-aware Autopilot [7]	21.6	23.4	**27.2**	21.4	21.9
PhyDAWS [10]	21.6	21.6	26.3	**23.5**	23.1
NTN [9]	20.4	21.2	24.4	20.4	20.7
**Ours**	21.7	**23.5**	25.1	20.3	22.8

No method ranks first across all four weather subsets. With SemanticKITTI as the source, WeatherMamba ranks among the top three for dense fog, light fog, rain, and snow, with mIoU values of 37.9%, 33.4%, 33.2%, and 31.9%, respectively. With SynLiDAR as the source, it ranks second for dense fog, first for light fog, third for rain, and fourth for snow. The 23.5% light-fog result is the highest in that panel, whereas the 20.3% snow result shows weaker performance in snow in this transfer setting. The overall mIoU of 22.8% ranks third behind UniMix [25] (23.4%) and PhyDAWS [10] (23.1%). The results, therefore, show a weather-dependent performance profile rather than uniform gains across conditions.

### 4.5. Source-Domain Performance Verification

We also evaluate WeatherMamba on the SemanticKITTI source domain to determine whether weather-robust refinement degrades clear-weather representations. In Table 3, WeatherMamba obtains 49.3% mIoU, 0.2 points below DarkNet53Seg [15], and the best result in eight classes. The full model, therefore, retains competitive source-domain performance while improving adverse-weather generalization.

### 4.6. Ablation and Sensitivity Analysis

Table 4, Table 5 and Table 6 use the same SemanticKITTI→SemanticSTF source-only protocol, source and target data splits, training schedule, and random seed. All settings remain fixed except for the module, evidence source, or augmentation-strength factor, which varies in each experiment.

Table 4 reports all module combinations. The backbone obtains 29.4% mIoU. Adding MANF, RADM, or WGRG individually increases mIoU to 31.8%, 30.2%, and 34.6%, respectively; WGRG provides the largest single-module gain of 5.2 points. MANF+WGRG reaches 36.3%, while the complete model achieves 37.4%. This is an 8.0-point improvement over the baseline and the highest mIoU in every weather condition among the tested combinations.

The two sensitivity studies vary one factor at a time. In Table 5, the support-preserving strength *s* and density/dropout strength *d* remain at the pre-specified defaults of 0.5 and 1.0, respectively, while the WGRG evidence source is varied. In Table 6, WGRG uses the combined intensity, density, and feature configuration. Panel (a) varies *s* with point dropout disabled and panel (b) fixes s=0.5 and varies *d*. All other model, data, training, and evaluation settings remain unchanged.

#### 4.6.1. WGRG Evidence Sensitivity

The WGRG evidence study follows the SemanticKITTI→SemanticSTF source-only protocol used in Table 4. MANF and RADM remain active, and only the evidence used to estimate degradation is varied.

Each observation-derived cue improves on the 32.6% reference. Feature evidence gives the strongest individual result at 36.7% mIoU, followed by intensity at 35.7% and density at 34.9%. Combining the three evidence sources yields 37.4% mIoU, 48.6% mAcc, and 71.6% allAcc. Improvements across all three metrics show that the gain is not limited to the most frequent classes: the combined setting improves both class-balanced and point-level accuracy. Its mIoU is 4.8 points higher than that of the reference configuration.

#### 4.6.2. Pseudo-Weather Strength Sensitivity

The pseudo-weather study follows the SemanticKITTI→SemanticSTF source-only protocol used in Table 4. MANF and RADM remain active, and each panel varies one augmentation factor.

Table 6 evaluates values around the pre-specified default strengths. In panel (a), s=0.5 gives the highest mIoU and mAcc, whereas s=1.0 gives the highest allAcc of 72.0%. In panel (b), d=1.0 gives the highest mIoU and mAcc, while d=0.5 gives the highest allAcc of 72.8%. The metrics favor different settings because allAcc aggregates predictions over all points and is influenced by frequent classes, whereas mIoU and mAcc weight classes equally. With mIoU as the primary metric, the pre-specified defaults s=0.5 and d=1.0 provide the strongest class-balanced results among the tested settings. Increasing the strengths to s=1.5 or d=1.5 reduces mIoU to 33.2% and 35.5%, respectively. In these experiments, stronger corruption, therefore, leads to lower segmentation performance.

### 4.7. Computational Efficiency Analysis

**Figure 6:**
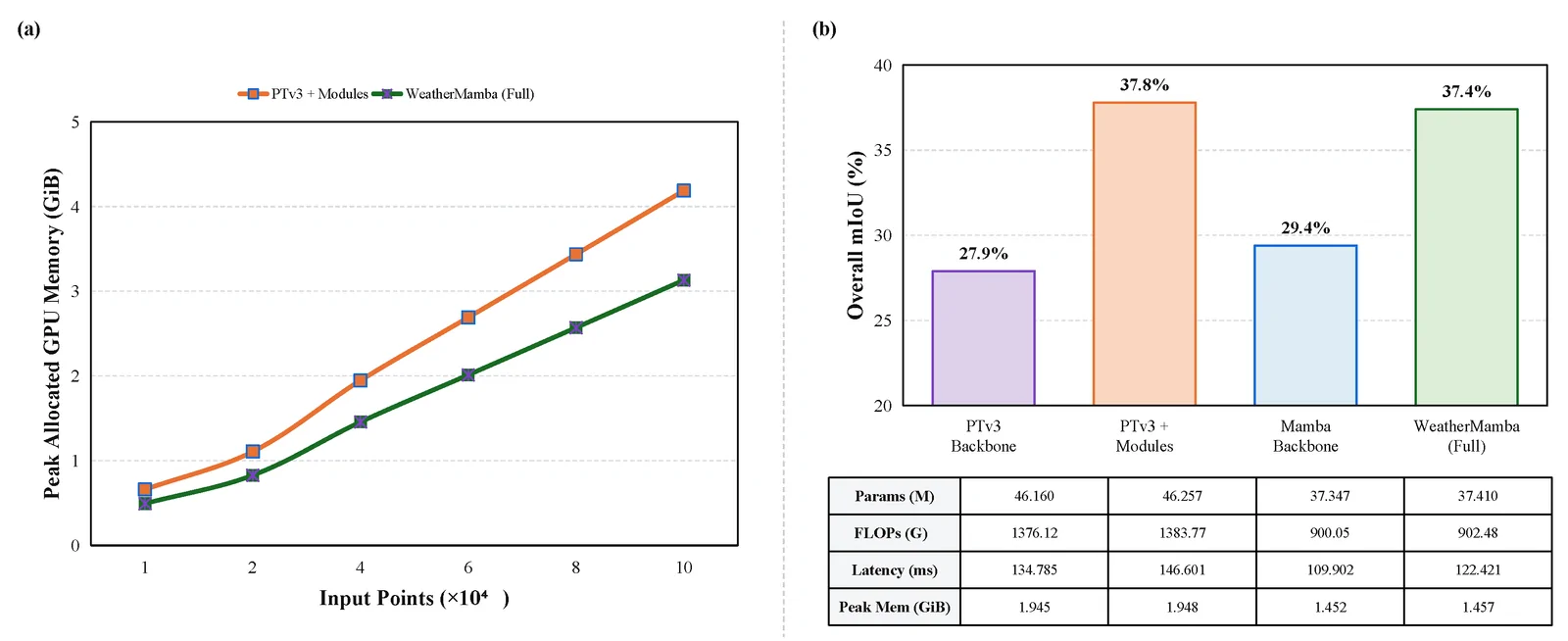
Accuracy–efficiency comparison of PTv3 and WeatherMamba: (**a**) peak allocated GPU memory across increasing input sizes; (**b**) mIoU and the parameters, FLOPs, latency, and peak allocated memory at the matched operating point.

Computational efficiency is measured in the same single-GPU FP32 environment, using a batch size of 4 and 8192 input points per scan at the matched operating point. All configurations follow the same measurement procedure. Figure 6 compares the Transformer-style PTv3 [11] reference with the Mamba-based WeatherMamba design. Across 10k–100k input points, WeatherMamba uses less peak allocated GPU memory than PTv3 with Modules. At 100k points, peak memory decreases from 4.188 to 3.132 GiB. At the matched operating point in Figure 6b, WeatherMamba uses 37.410 M parameters, 902.48 G FLOPs, 122.421 ms latency, and 1.457 GiB peak allocated memory. PTv3 with Modules uses 46.257 M parameters, 1383.77 G FLOPs, 146.601 ms latency, and 1.948 GiB. These differences correspond to reductions of 19.1%, 34.8%, 16.5%, and 25.2% in parameters, FLOPs, latency, and peak memory, respectively, with a 0.4-point lower mIoU (37.4% versus 37.8%). Relative to the Mamba backbone, the complete model adds 0.063 M parameters and 2.43 G FLOPs while increasing mIoU from 29.4% to 37.4%. WeatherMamba, therefore, offers a favorable accuracy–efficiency operating point relative to the matched PTv3 reference, but not an across-the-board accuracy advantage.

### 4.8. Visualization Analysis

**Figure 7:**
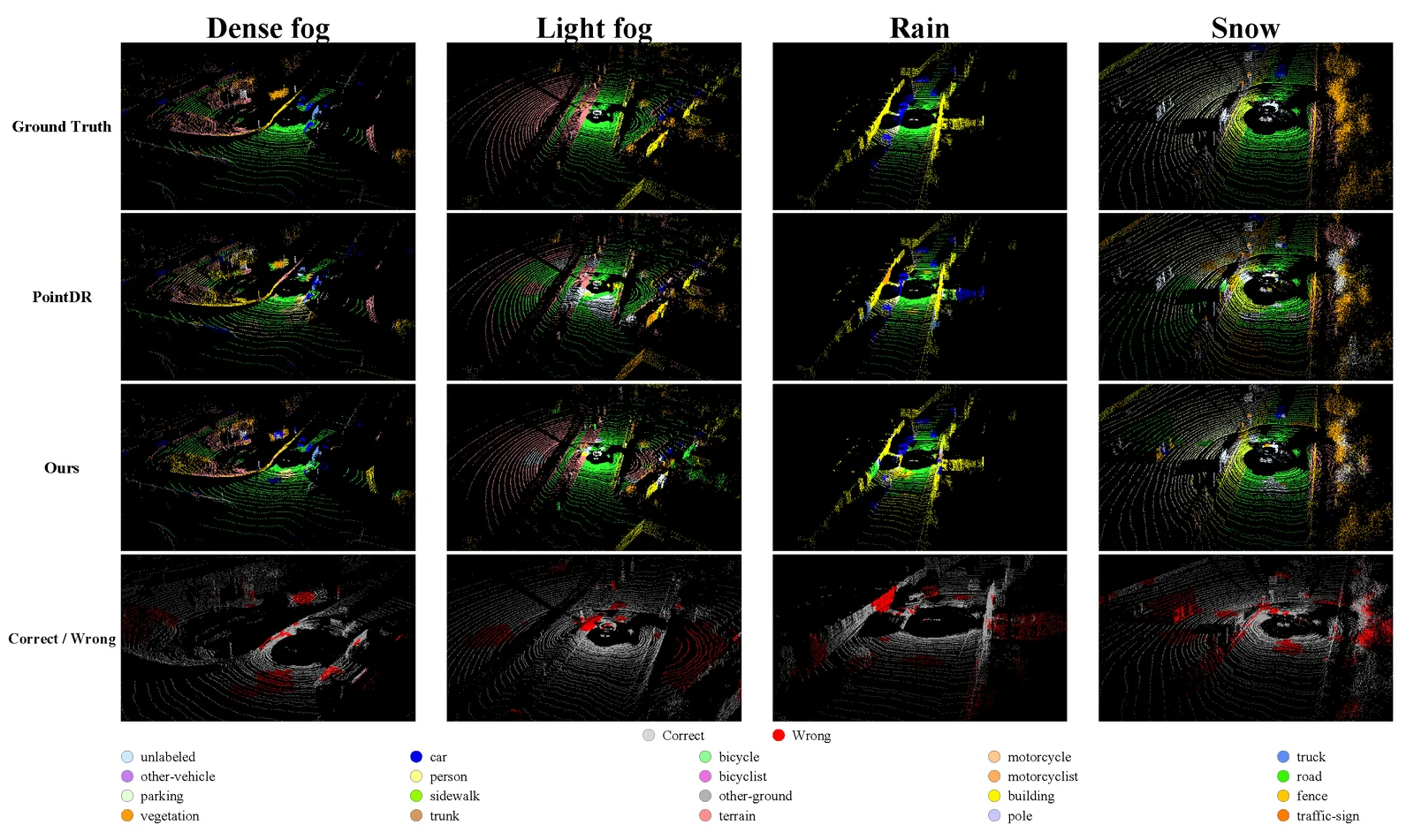
Qualitative segmentation comparison under adverse weather conditions.

Figure 7 compares PointDR [6] and WeatherMamba under dense fog, light fog, rain, and snow conditions using a shared semantic color legend. In the highlighted regions, WeatherMamba produces more continuous object boundaries and fewer scattered false predictions.

**Figure 8:**
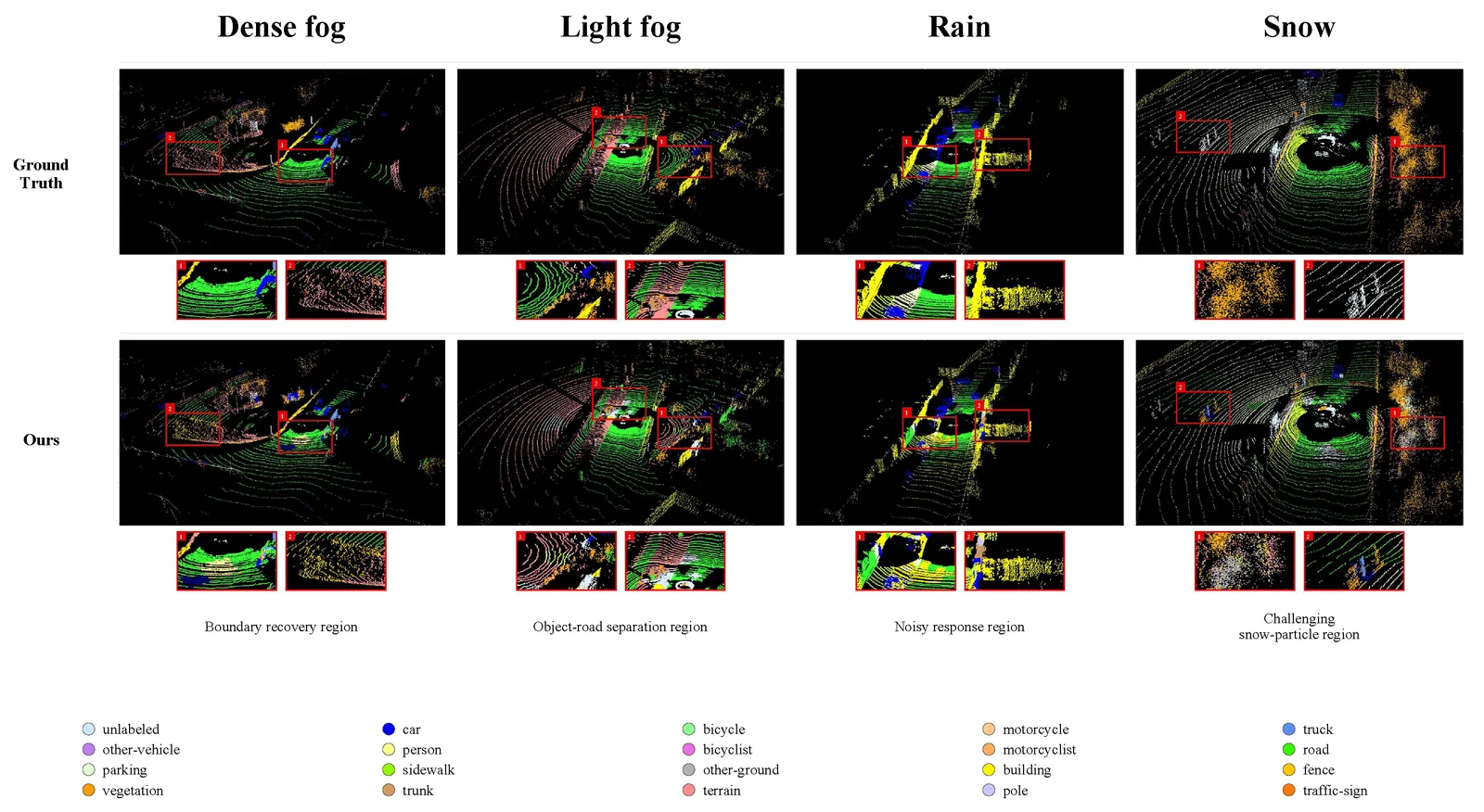
Enlarged qualitative comparison under adverse weather conditions.

Figure 8 enlarges representative local regions to show boundary recovery, object–road separation, and the suppression of noisy responses under dense fog, light fog, rain, and snow conditions.

## 5. Discussion

WeatherMamba assigns distinct functions to four components within a source-only framework. MANF aggregates multiscale local support, RADM conditions feature refinement on point-wise reliability, the Mamba backbone propagates long-range context, and WGRG gates bound residual corrections using observation-derived evidence. In the single-seed ablation, the complete model improves the module-free baseline from 29.4% to 37.4% mIoU and gives the highest result among the tested module combinations (Table 4). This result is consistent with complementary contributions from the components, although a single-seed ablation cannot establish their internal mechanisms independently.

The sensitivity experiments characterize WGRG evidence and the pre-specified pseudo-weather defaults within the tested ranges. Feature evidence gives the highest individual mIoU of 36.7%. Combining intensity, density, and feature evidence yields 37.4% mIoU, 48.6% mAcc, and 71.6% allAcc (Table 5). The pre-specified strengths s=0.5 and d=1.0 also give the highest mIoU in their respective panels, whereas the larger tested strengths reduce mIoU. These results favor moderate source-domain corruption over stronger perturbation for the evaluated setting.

WeatherMamba is competitive but does not achieve state-of-the-art aggregate accuracy. Under the SemanticKITTI-source protocol, it trails NTN [9] by 0.8 points and PhyDAWS [10] by 0.5 points. Under the SynLiDAR-source protocol, it trails UniMix [25] by 0.6 points and PhyDAWS [10] by 0.3 points. Its value lies in the combined profile of results: the highest IoU in five classes under each transfer protocol, observation-only WGRG inference without weather metadata or pre-known target reflectance, and lower parameters, FLOPs, latency, and peak memory than PTv3 with Modules [11], with a 0.4-point mIoU trade-off. Under the evaluated adverse-weather transfer settings, WeatherMamba is, therefore, a resource-conscious alternative with class-specific strengths.

The evaluation has three limitations. First, the ablation and sensitivity studies use a single random seed; multi-seed evaluation is needed to quantify statistical variation. Second, snow remains difficult because small-object structures and weather particles can produce similar sparse local patterns. Third, pseudo-weather augmentation represents sensor-relevant degradation directions but not the complete atmospheric radiative-transfer process. Future work will examine multi-seed stability, cross-sensor transfer, and physically calibrated weather simulation.

## 6. Conclusions

WeatherMamba addresses domain-generalized adverse-weather LiDAR point-cloud semantic segmentation through an observation-derived continuous point-wise gate and dedicated pathways for multiscale local support, reliability-conditioned feature refinement, and long-range state-space context. The gate requires no externally supplied weather type, pre-known target reflectance, or deployment-time meteorological input. WeatherMamba achieves 37.4% mIoU on SemanticKITTI→SemanticSTF and 22.8% on SynLiDAR→SemanticSTF, with the highest class IoU in five classes under each transfer direction. The complete configuration improves the module-free baseline by 8.0 points, and combined WGRG evidence improves its reference by 4.8 points. Relative to PTv3 with Modules, WeatherMamba reduces parameters, FLOPs, latency, and peak allocated memory with a 0.4-point mIoU trade-off. The results support competitive accuracy and efficiency under observation-only deployment, while broader statistical and cross-sensor validation remains necessary.

## Figures and Tables

**Table 1:** Class-wise domain-generalization comparison under SemanticKITTI→SemanticSTF and SynLiDAR→SemanticSTF. All IoU values are reported in %; the best and second-best results in each column are shown in bold and underlined, respectively.

Method	Car	Bi.cle	Mt.cle	Truck	Oth-v	Pers	Bi.clst	Mt.clst	Road	Parki	Sidew	Oth-g	Build	Fence	Veget	Trunk	Terra	Pole	Traf	mIoU (%)
SemanticKITTI→SemanticSTF																				
Dropout [28]	62.1	0.0	15.5	3.0	11.5	5.4	2.0	0.0	58.4	12.8	26.7	1.1	72.1	43.6	52.9	34.2	43.5	28.4	15.5	25.7
PolarMix [24]	57.8	1.8	3.8	16.7	3.7	26.5	0.0	2.0	65.7	2.9	32.5	0.3	71.0	48.7	53.8	20.5	45.4	25.9	15.8	26.0
PointDR [6]	67.3	0.0	4.5	19.6	9.0	18.8	2.7	0.0	62.6	12.9	38.1	0.6	73.3	43.8	56.4	32.2	45.7	28.7	27.4	28.6
DGUIL [29]	77.9	**10.6**	19.1	26.0	9.7	46.3	6.0	9.3	**69.1**	18.0	38.6	9.4	73.3	51.2	60.8	30.9	50.8	31.8	22.3	34.7
UniMix [25]	**82.7**	6.6	8.6	4.5	15.1	35.5	**15.5**	37.7	55.8	10.2	36.2	1.3	72.8	40.1	49.1	33.4	34.9	23.5	33.5	31.4
DGLSS [30]	72.6	0.1	11.7	29.4	13.7	48.3	0.5	21.2	65.0	**20.2**	38.3	3.8	**78.9**	51.8	57.0	36.4	47.0	26.9	34.9	34.6
Weather-aware Autopilot [7]	72.0	0.0	32.9	37.0	1.9	37.7	6.8	52.9	59.9	10.7	31.8	2.2	76.0	48.8	**62.7**	34.0	49.3	23.6	20.4	34.8
PhyDAWS [10]	64.3	0.8	**49.9**	**37.2**	10.3	44.2	5.4	**57.9**	63.7	18.5	34.8	3.9	76.6	49.2	55.4	**39.0**	46.5	28.3	34.3	37.9
NTN [9]	81.9	3.6	30.8	35.6	**17.9**	**52.4**	6.7	54.9	66.0	17.8	36.6	5.3	70.9	41.1	57.0	35.4	45.2	27.7	**39.1**	**38.2**
**Ours**	75.5	3.2	16.5	27.8	14.5	47.2	12.0	20.5	68.5	18.2	**42.3**	**25.0**	74.5	**54.8**	62.2	34.5	**50.9**	**32.6**	29.9	37.4
SynLiDAR→SemanticSTF																				
Dropout [28]	28.0	3.0	1.4	9.6	0.0	17.1	0.8	0.7	34.2	6.8	19.1	0.1	35.5	19.1	42.3	17.6	36.0	14.0	2.8	15.2
PolarMix [24]	39.2	1.1	1.2	8.3	1.5	17.8	0.8	0.7	23.3	1.3	17.5	0.4	45.2	24.8	46.2	20.1	38.7	7.6	1.9	15.7
PointDR [6]	37.8	2.5	2.4	23.6	0.1	26.3	2.2	3.3	27.9	7.7	17.5	0.5	47.6	25.3	45.7	21.0	37.5	17.9	5.5	18.5
DGUIL [29]	43.3	2.8	2.6	23.2	3.2	31.3	2.5	4.4	34.3	9.2	17.9	0.3	57.1	27.6	50.0	24.2	41.5	19.0	6.1	21.1
UniMix [25]	**65.4**	0.1	**3.9**	16.9	**5.3**	32.3	2.0	**19.3**	52.1	5.0	**27.3**	**3.0**	49.4	20.3	58.5	22.7	23.2	26.9	10.4	**23.4**
DGLSS [30]	47.9	2.9	3.4	17.4	1.1	28.0	2.4	7.3	28.8	10.2	18.1	0.2	48.9	25.3	46.5	21.4	45.2	17.9	4.9	19.8
Weather-aware Autopilot [7]	33.8	1.1	2.9	17.0	0.2	26.8	1.0	4.3	**53.9**	5.0	20.6	2.2	64.3	27.1	53.8	**27.0**	37.0	**28.6**	8.6	21.9
PhyDAWS [10]	46.9	**3.8**	2.0	18.4	0.7	**34.7**	3.4	5.6	37.9	9.7	20.2	0.9	**69.3**	**31.5**	53.7	26.4	39.7	22.5	**11.4**	23.1
NTN [9]	47.5	1.5	2.4	19.0	0.2	28.5	3.1	8.7	42.7	6.6	20.1	0.0	51.2	29.5	48.8	19.6	32.3	24.2	7.4	20.7
**Ours**	50.5	3.0	3.4	**27.5**	1.2	30.1	**4.1**	6.4	34.2	**12.2**	22.8	1.4	63.0	30.8	**60.0**	26.8	**49.5**	3.0	3.4	22.8

**Table 3:** Segmentation results for the SemanticKITTI source domain. All IoU values are reported in %; the best and second-best results in each column are shown in bold and underlined, respectively.

Method	Road	Sidewalk	Parking	Oth-g	Build	Car	Truck	Bicycle	Mt.cle	Oth-v	Veget	Trunk	Terra	Pers	Bi.cle	Mt.clst	Fence	Pole	Traf	mIoU (%)
PointNet [31]	61.6	35.7	15.8	1.4	41.4	46.3	0.1	1.3	1.3	0.8	31.0	4.6	17.6	0.2	0.2	0.0	12.9	2.4	3.7	14.6
SPGraph [32]	45.0	28.5	0.6	0.6	64.3	49.3	0.1	0.2	0.2	0.8	48.9	27.2	24.6	0.3	2.7	0.1	20.8	15.9	0.8	17.4
SPLATNet [33]	64.6	39.1	0.4	0.0	58.3	58.2	0.0	0.0	0.0	0.0	71.1	9.9	19.3	0.0	0.0	0.0	23.1	5.6	0.0	18.4
PointNet++ [12]	72.0	41.8	18.7	5.6	62.3	53.7	0.9	1.9	1.9	0.2	46.5	13.8	30.0	0.9	1.0	0.0	16.9	6.0	8.9	20.1
SqueezeSeg [34]	85.4	54.3	26.9	4.5	57.4	68.8	3.3	16.0	16.0	3.6	60.0	24.3	53.7	12.9	13.1	0.9	29.0	17.5	24.5	29.5
SqueezeSegV2 [35]	88.6	67.6	45.8	17.7	73.7	81.8	13.4	18.5	18.5	14.0	71.8	35.8	60.2	20.1	25.1	3.9	41.1	20.2	36.3	39.7
TangentConv [36]	83.9	63.9	33.4	15.4	83.4	**90.8**	15.2	2.7	2.7	12.1	**79.5**	49.3	58.1	23.0	28.4	**8.1**	49.0	35.8	28.5	40.9
DarkNet21Seg [37]	91.4	74.0	57.0	26.4	81.9	85.4	18.6	26.2	26.2	15.6	77.6	48.4	63.6	31.8	33.6	4.0	52.3	36.0	50.0	47.4
**Ours**	85.9	**79.7**	57.0	23.9	83.5	87.2	24.7	**27.6**	**29.3**	**23.8**	73.9	48.9	**65.0**	35.3	**36.9**	5.6	53.6	**41.7**	**53.2**	49.3
DarkNet53Seg [15]	**91.8**	74.6	**64.8**	**27.9**	**84.1**	86.4	**25.5**	24.5	24.5	22.6	78.3	**50.1**	64.0	**36.2**	33.6	4.7	**55.0**	38.9	52.2	**49.5**

**Table 4:** Weather-wise module ablation under SemanticKITTI→SemanticSTF. All values are mIoU (%); the final mIoU column reports performance on the complete SemanticSTF validation set rather than the unweighted mean of the four weather subsets. The two highest results in each column are shown in bold. Here, ✓ denotes that the corresponding module is enabled, whereas – denotes that it is disabled.

MANF	RADM	WGRG	Dense Fog	Light Fog	Rain	Snow	mIoU (%)
–	–	–	31.2	27.4	29.1	24.6	29.4
✓	–	–	33.5	29.2	30.8	27.1	31.8
–	✓	–	32.3	28.0	30.0	26.0	30.2
–	–	✓	35.9	31.3	32.2	29.8	34.6
✓	✓	–	34.6	29.8	31.7	28.5	32.6
✓	–	✓	**37.1**	**32.6**	**32.8**	**31.1**	**36.3**
–	✓	✓	34.8	30.1	31.6	28.7	32.9
✓	✓	✓	**37.9**	**33.4**	**33.2**	**31.9**	**37.4**

**Table 5:** WGRG evidence sensitivity under the same SemanticKITTI→SemanticSTF protocol as Table 4. MANF and RADM remain active, and all settings other than the tested evidence source are fixed. Metrics are reported in %; Δ mIoU is measured relative to the reference configuration. The two highest results in each metric are shown in bold. For the reference configuration, – indicates that a relative improvement is not applicable.

Configuration	Effective Evidence	mIoU (%)	mAcc (%)	allAcc (%)	Δ mIoU (pp)
Reference	None	32.6	43.6	67.8	–
WGRG-intensity	Intensity	35.7	45.9	69.7	+3.1
WGRG-density	Density	34.9	45.2	68.9	+2.3
WGRG-feature	Feature	**36.7**	**47.4**	**70.8**	**+4.1**
**WGRG-all**	Intensity+density+feature	**37.4**	**48.6**	**71.6**	**+4.8**

**Table 6:** Pseudo-weather strength sensitivity under the same SemanticKITTI→SemanticSTF protocol as Table 4. MANF and RADM remain active, and all settings other than the tested corruption strength are fixed. Panel (a) varies support-preserving corruption with point dropout disabled ($p_{\mathrm{drop}}=0$); panel (b) varies density/dropout corruption with support-preserving strength fixed at 0.5. Metrics are reported in %; the best result in each metric is shown in bold. In each panel, – in the Δ mIoU column denotes the reference setting, for which no relative change is reported.

(a) Support-preserving corruption				
Strength *s*	mIoU (%)	mAcc (%)	allAcc (%)	Δ mIoU (pp)
0.0	35.8	48.0	70.3	–
**0.5**	**37.4**	**48.6**	71.6	**+1.6**
1.0	35.7	47.9	**72.0**	−0.1
1.5	33.2	44.8	68.8	−2.6
**(b) Density/dropout corruption**				
Strength *d*	mIoU (%)	mAcc (%)	allAcc (%)	Δ mIoU (pp)
0.5	35.8	47.6	**72.8**	–
**1.0**	**37.4**	**48.6**	71.6	**+1.6**
1.5	35.5	47.2	72.2	−0.3

## Data Availability

The datasets used in this study are publicly available from their respective official sources. SemanticKITTI, SynLiDAR and SemanticSTF can be accessed from their official project pages or repositories. Processed splits and experimental configurations can be made available upon reasonable request. The source code is currently being organized and documented and will be released in a public repository after this process is completed.

## Abbreviations

The following abbreviations are used in this manuscript:

LiDAR	Light Detection and Ranging
MANF	Multiscale Adaptive Neighborhood Fusion
RADM	Reliability-Aware Denoising Module
WGRG	Weather-Guided Reliability Gating
SSM	State-space model
mIoU	Mean Intersection over Union
